# The RhoGEF TEM4 Regulates Endothelial Cell Migration by Suppressing Actomyosin Contractility

**DOI:** 10.1371/journal.pone.0066260

**Published:** 2013-06-18

**Authors:** Natalia Mitin, Kent L. Rossman, Rachel Currin, Sandeep Anne, Thomas W. Marshall, James E. Bear, Victoria L. Bautch, Channing J. Der

**Affiliations:** 1 Lineberger Comprehensive Cancer Center, University of North Carolina, Chapel Hill, North Carolina, United States of America; 2 Department of Pharmacology, University of North Carolina, Chapel Hill, North Carolina, United States of America; 3 Department of Cell and Developmental Biology, University of North Carolina, Chapel Hill, North Carolina, United States of America; 4 Howard Hughes Medical Institute, University of North Carolina, Chapel Hill, North Carolina, United States of America; 5 Department of Biology, University of North Carolina, Chapel Hill, North Carolina, United States of America; 6 McAllister Heart Institute, University of North Carolina, Chapel Hill, North Carolina, United States of America; Leiden University, Netherlands

## Abstract

Persistent cellular migration requires efficient protrusion of the front of the cell, the leading edge where the actin cytoskeleton and cell-substrate adhesions undergo constant rearrangement. Rho family GTPases are essential regulators of the actin cytoskeleton and cell adhesion dynamics. Here, we examined the role of the RhoGEF TEM4, an activator of Rho family GTPases, in regulating cellular migration of endothelial cells. We found that TEM4 promotes the persistence of cellular migration by regulating the architecture of actin stress fibers and cell-substrate adhesions in protruding membranes. Furthermore, we determined that TEM4 regulates cellular migration by signaling to RhoC as suppression of RhoC expression recapitulated the loss-of-TEM4 phenotypes, and RhoC activation was impaired in TEM4-depleted cells. Finally, we showed that TEM4 and RhoC antagonize myosin II-dependent cellular contractility and the suppression of myosin II activity rescued the persistence of cellular migration of TEM4-depleted cells. Our data implicate TEM4 as an essential regulator of the actin cytoskeleton that ensures proper membrane protrusion at the leading edge of migrating cells and efficient cellular migration via suppression of actomyosin contractility.

## Introduction

Cellular migration plays a critical role in many physiological and pathological processes including normal cell embryogenesis, wound healing, and tumor cell metastasis. Migrating cells advance by extending their front and retracting their rear [1]. Protrusion of the cell front (leading edge) is regulated by continuous remodeling of the actin cytoskeleton and formation of F-actin filaments crosslinked with myosin II [2], [3], [4], [5]. The assembly of actin filaments into an actomyosin-crosslinked contractile network is essential for membrane protrusion and whole cell migration [2], [5], [6]. However, myosin II-driven contractility at the cell front is tightly regulated during cell migration to ensure leading edge advance, as inhibition of myosin II activity promotes leading edge protrusion [5], [7], [8], [9].

Rho family GTPases are key regulators of actin cytoskeleton dynamics [1], [10]. Biochemically, Rho GTPases are molecular switches that cycle dynamically between inactive, GDP-bound and active, GTP-bound states [11]. Guanine nucleotide exchange factors for Rho GTPases (RhoGEFs) catalyze the exchange of bound GDP for GTP to favor formation of Rho-GTP and activation of downstream effector functions [12], [13]. The largest family of RhoGEFs in humans is the Dbl family of proteins [12]. Dbl family proteins are characterized by a tandem catalytic Dbl homology (DH) and regulatory pleckstrin homology (PH) domain cassette responsible for accelerating the intrinsic nucleotide exchange activity of Rho GTPases.

*TEM4* (tumor endothelial marker 4) was identified originally as a gene whose expression was upregulated in endothelial cells during tumor cell-induced angiogenesis [14]. Recently, we have shown that TEM4 is a Rho-specific guanine nucleotide exchange factor (GEF) and a member of Dbl family of RhoGEFs [12], [15]. However, the role of TEM4 in endothelial cell biology remains to be determined.

Here, we show that TEM4 regulates endothelial cell migration. Specifically, TEM4 signaling is essential to maintain the organization of the actin cytoskeleton and focal adhesions in protrusive areas of the cell. We show that TEM4 mediates its function, at least in part, by suppressing actomyosin contractility. Our data implicate TEM4 as an essential regulator of the actin cytoskeleton to ensure proper membrane protrusion of the leading edge and efficient endothelial cell migration.

## Results

### TEM4 is a Regulator of Angiogenesis

**Figure 1:**
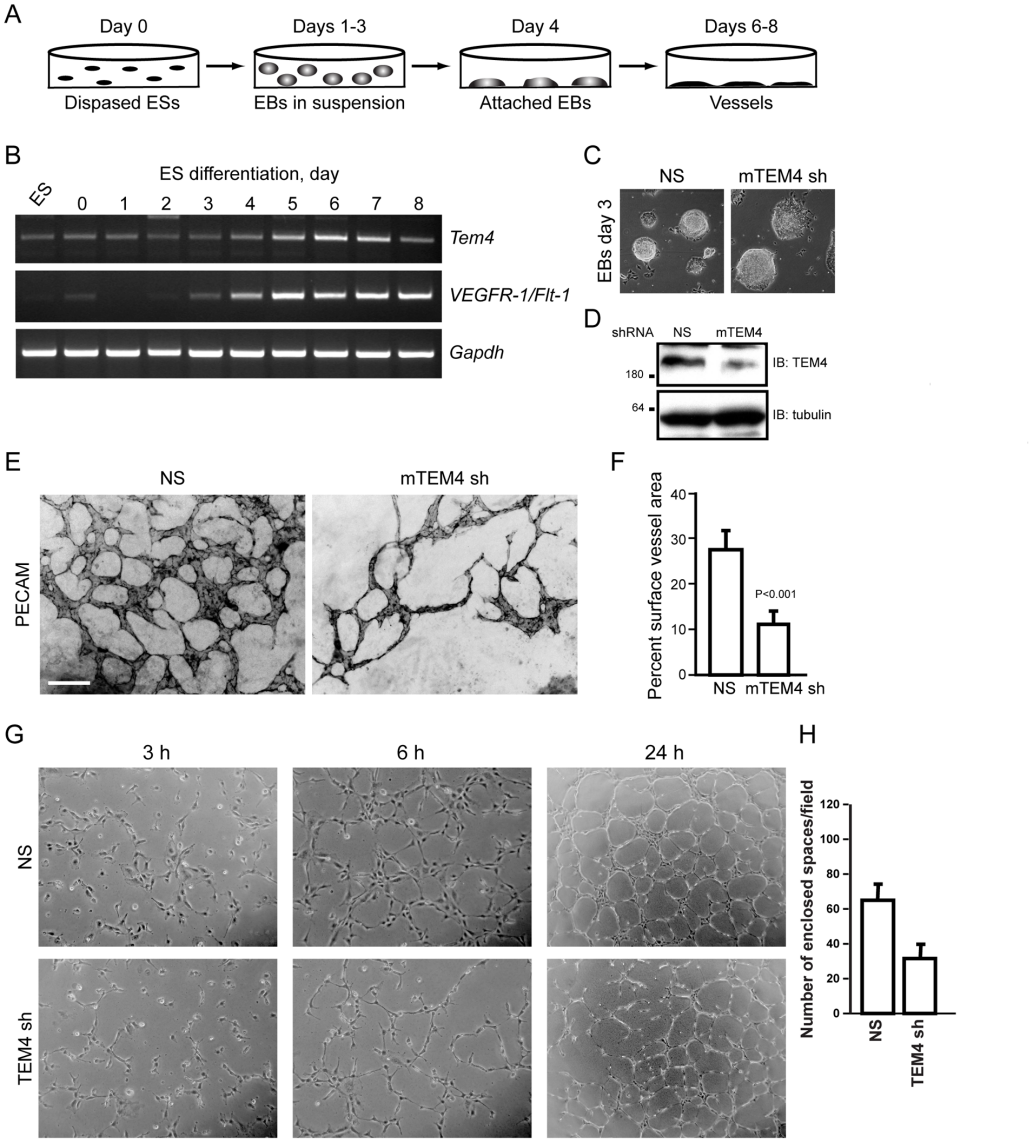
TEM regulates blood vessel formation. (A) Cartoon describing differentiation of ES cells to form blood vessels. EBs, embryoid bodies. (B) RT-PCR analysis of *Tem4*, *VEGFR-1* and *Gapdh* expression during differentiation of ES cells. ES, embryonic stem cells prior to dispersion by dispase treatment. Data are representative of two independent experiments. (C) Knockdown of TEM4 does not inhibit differentiation of ES cells into EBs. (D) Western blot confirming knockdown of TEM4 in ES cells by lentivirus-based RNAi construct. (E, F) Loss of TEM4 impairs blood vessel formation. Differentiated ES cell cultures were fixed on day 8 and vessels visualized by staining with PECAM (E). PECAM-positive surface area was measured and graphed (F) as described in Materials and methods. Scale bar, 200 µm. (G) Loss of TEM4 impairs Matrigel tubule formation in vitro. Phase contrast images of HUVECs expressing NS control or TEM4 shRNA undergoing in vitro tubulogenesis on Matrigel surface. (H) Quantitation of Matrigel tubule formation assay (n = 3, 4 fields/well).

The identification of TEM4 in a screen for regulators of angiogenesis *in vivo* [14], where *TEM4* expression was found to be upregulated in tumor vasculature of colorectal cancer patients, suggested that this RhoGEF may serve an important role during angiogenesis. To determine whether TEM4 is involved in angiogenesis, we utilized a mouse embryonic stem (ES) cell differentiation model of vascular development. Mouse ES cells, derived from the inner cell mass of blastocyst stage embryos, undergo programmed differentiation *in vitro* to form a primitive vasculature that closely resembles early vascular development *in vivo* [16], [17], [18], [19], [20]. In this assay, aggregated ES cells are partially differentiated in suspension to form embryoid bodies (EBs) (Fig. 1A, day 1–3) which are then replated for attachment (day 3). During the next several days, EBs differentiate into multiple cell types, including endothelial cells that proliferate and migrate to form primitive vessels, giving rise to a highly branched vascular network [19], [21], [22]. Using RT-PCR analysis, we found that *Tem4* expression increased during development of ES cell–derived vessels (Fig. 1B; Days 5–7). As expected, expression of VEGFR-1 appeared at day 4 and persisted throughout ES cell differentiation [23]. These data indicate that Tem4 is upregulated during vascular development in the ES model of angiogenesis, which is consistent with TEM4 expression during angiogenesis *in vivo* [14]. Knockdown of Tem4 during ES cell differentiation did not impair ES cell growth or formation of EBs (Fig. 1C). However, the loss of Tem4 severely impaired blood vessel formation, decreasing total vessel surface area and branching (Fig. 1, E and F). These results suggest that TEM4 is essential for angiogenesis.

To further examine a possible role of TEM4 in angiogenesis, we performed Matrigel tubulogenesis assay on human umbilical vein primary endothelial cells (HUVECs). When plated on Matrigel, endothelial cells spread on the Matrigel, migrate towards each other and fuse to form pre-capillary cords closely resembling the *in vivo* vasculature [24], [25], [26]. Monitoring cells undergoing tubulogenesis on the Matrigel would allow us to identify distinct steps at which TEM4 may impact *in vivo* angiogenesis. As shown in Figure 1G, within 3–6 h of plating control cells migrated towards each other and began aligning to form vascular webs. On the contrary, TEM4-depleted cells lagged behind and failed to form complex webs seen in control cells even by 24 h (Fig. 1H). Defects in tubulogenesis of TEM4-depleted cells suggest that TEM4 may regulate the most fundamental cellular processes such as cellular adhesion and migration.

### TEM4 and RhoC Regulate Persistence of Endothelial Cell Migration

**Figure 2:**
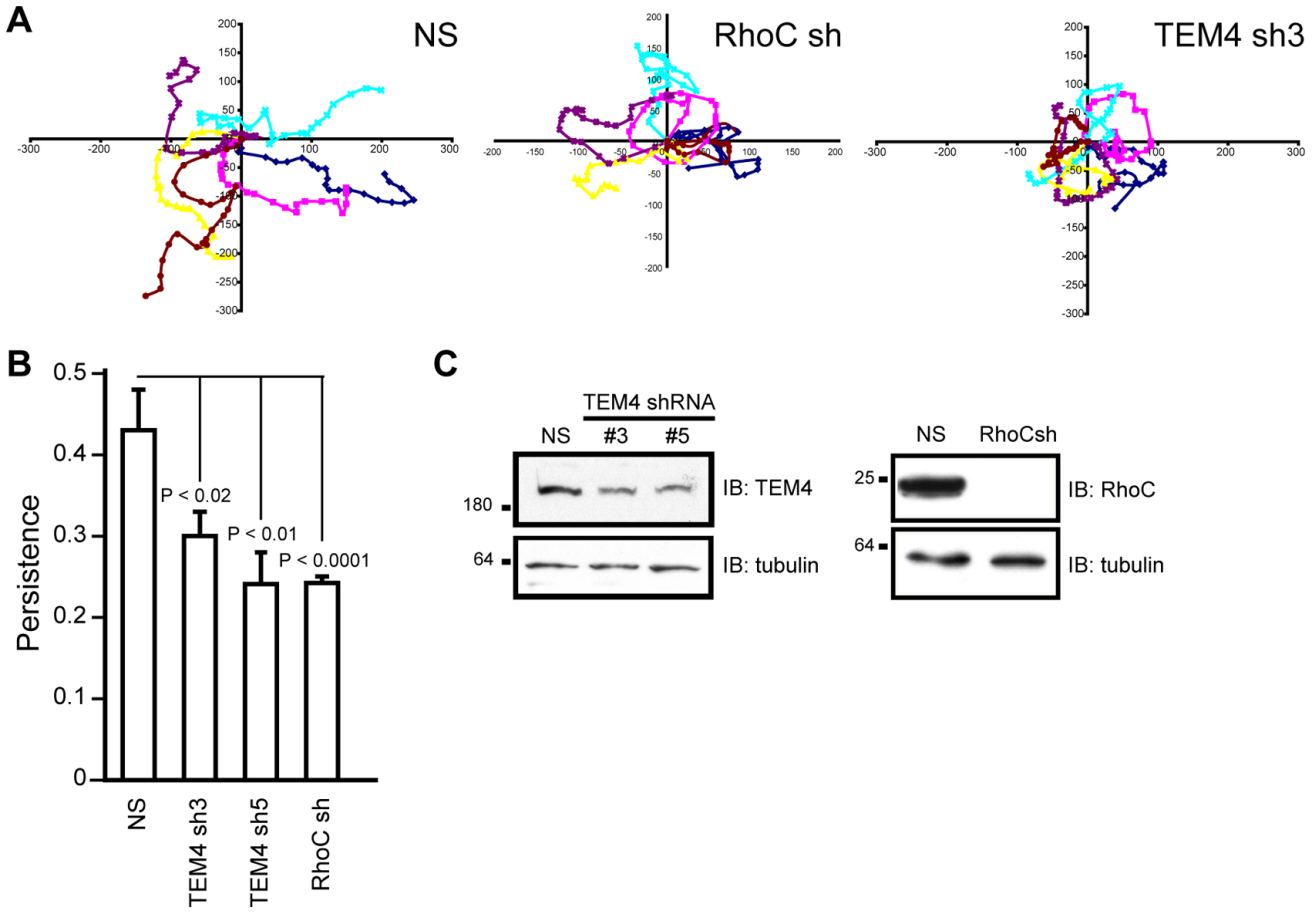
TEM4 regulates persistence of endothelial cell migration. (A) Wind-Rose plots depicting migratory tracks of six individual migrating cells in each experimental group. Values on x and y scales are arbitrary. (B) Persistence of two-dimensional cellular migration of HUVECs expressing NS, TEM4, or RhoC shRNAs. Data shown are the mean ± s.e.m. measured from 18–27 individual cells in 3–5 independent experiments. Cells expressing plasmid-based shRNA constructs were identified by monitoring GFP or mCherry fluorescence (Fig. S1). (C) Western blot confirming knockdown of TEM4 and RhoC expression levels by lentivirus-based RNAi constructs. NS; non-specific shRNA.

To determine if TEM4 regulates endothelial cell migration, we monitored cellular motility of HUVECs depleted of TEM4 by shRNA. From these analyses, we observed that cells depleted of TEM4 made frequent 90° turns and consequently, they inefficiently migrated away from the point of origin when compared to control cells expressing non-specific (NS) shRNA (Fig. 2A, Movie S1–S2). As we previously identified RhoC as an *in vivo* target of TEM4, we determined if RhoC has a function in endothelial cell migration. Cells depleted of RhoC exhibited a similar loss of directionality of migration phenotype as was seen with TEM4 depletion (Fig. 2A, Movie S3). Consistent with our visual observations, we found that persistence of migration (calculated as net displacement from origin/total length of migration path) was significantly decreased in cells with decreased TEM4 or RhoC expression levels (Fig. 2B–C, Fig. S1), suggesting that TEM4 regulates persistence of endothelial cell migration in part through RhoC activation.

### TEM4 Activates RhoC in Protruding Membranes

**Figure 3:**
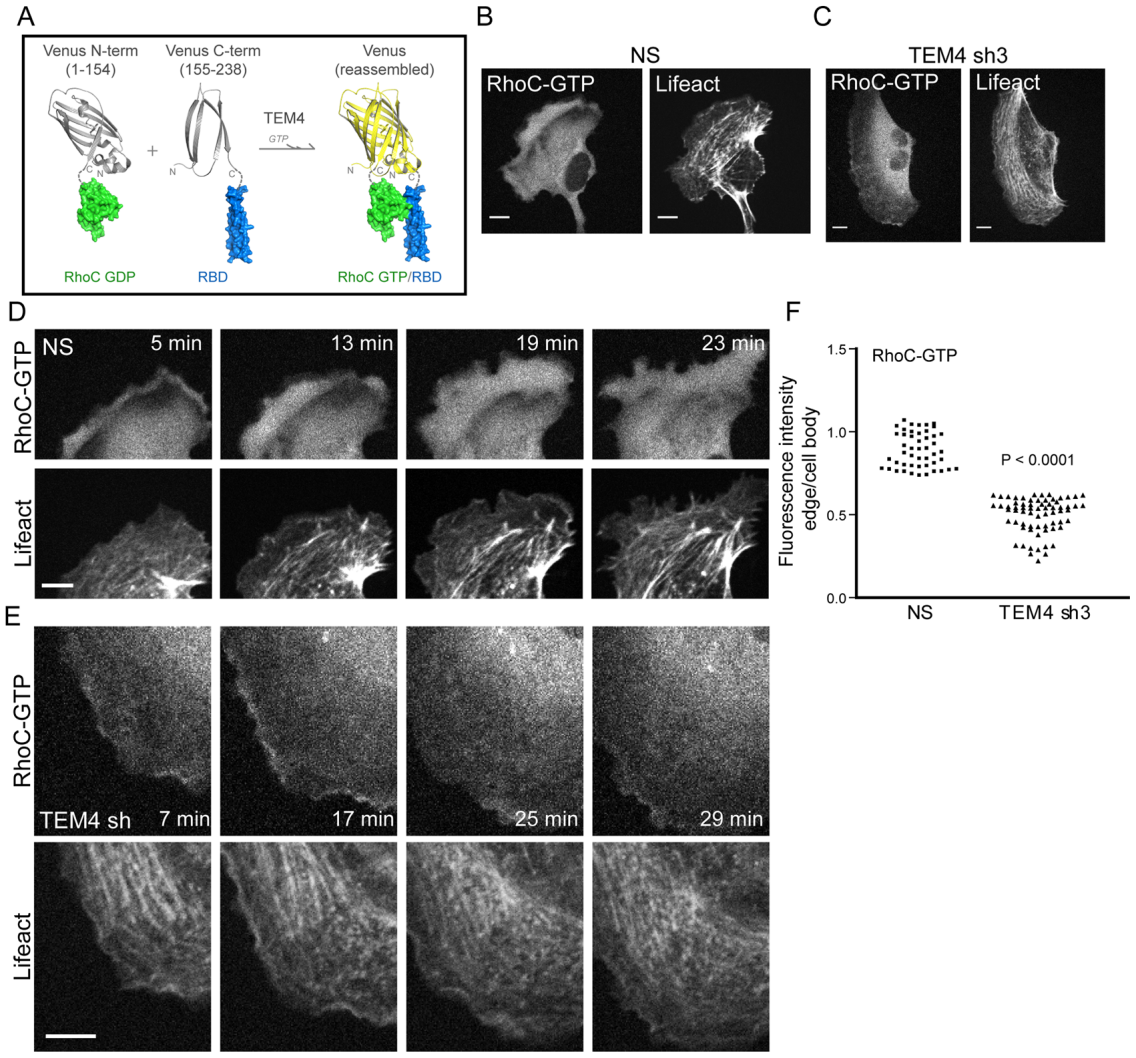
TEM4 activates RhoC in migrating cells. (A) Schematic diagram representing the principle of the BiFC assay. N- and C-terminal fragments of Venus fluorescent protein were fused to wild type RhoC and ROCKI RBD, respectively. An interaction between active, GTP-loaded RhoC and ROCK would facilitate association between N and C termini of Venus to produce a bimolecular fluorescent complex. (B–D) Activation of RhoC seen by BiFC assay in HUVECs expressing NS control (B, D) or TEM4 shRNA (C, E) and Lifeact-tRFP fusion protein to visualize actin. The close-up of the protrusive region demonstrates activation of RhoC in NS control (D) or TEM4-depleted cells (E). (F) Relative fluorescence intensity of areas at the leading edge as compared to areas within cell body in NS and TEM4-depleted cells. Measurements were performed as described in Methods for 4–5 protrusions throughout the time-lapse in 5 cells each. Scale bar, 10 µm.

We next determined if TEM4-dependent migration was associated with RhoC activation. A previous study suggested that RhoC is activated in membrane protrusions at the leading edge [27]. We therefore determined if activation of RhoC in membrane protrusions of endothelial cells is impaired in cells depleted of TEM4. To visualize activation of RhoC in membrane protrusions, we utilized a bimolecular fluorescence complementation (BiFC) assay, a widely accepted approach for studying spatial localization of protein-protein interactions [28], [29] and, more recently, utilized to study activation of the Ras GTPase protein [30]. Using available structural data [31], [32], we designed a BiFC probe for RhoC activation by fusing wild-type full-length RhoC to the N-terminus of Venus (VN; residues 1–154), a spectral derivative of the GFP protein [33], and the Rho-binding domain (RBD) of ROCKI (residues 947–1015) to a Venus C-terminal fragment (VC; residues 155–238) (Fig. 3 A and Fig. S2A). Functional fluorescent Venus protein is then reconstituted from the separate N- and C-terminal fragments [34] upon the specific binding of activated GTP-bound RhoC to the RBD (Fig. 3 A). Because wild-type RhoC protein is used in Venus fusion, it must be GTP-loaded by a RhoGEF to promote RBD binding. Therefore, the RhoC-BiFC probe can be used to monitor activation of RhoC *in vivo*. We have validated the specificity of RhoC-BiFC probe (see Results S1 and Figs. S2–S3) and concluded that the BiFC-based sensor can be used to visualize the spatial activation of RhoC in cells.

To determine if TEM4 promotes activation of RhoC in membrane protrusions, we visualized activation of RhoC in control NS and TEM4-depleted cells using spinning disk confocal microscopy. The F-actin marker, Lifeact [35] was used along with BiFC sensor to ensure that all movies were collected in the same focal plane towards the bottom of each cell. As we speculated, an increase in BiFC signal indicating activation of RhoC was detected in protruding membranes in NS control cells (Fig. 3B, D, F and Movie S4). More importantly, TEM4 knockdown severely impaired RhoC activation in protruding membranes (Fig. 3B, E, F and Movie S5) indicating TEM4-dependent RhoC activation. Although the role of RhoC in endothelial cell migration has not been established, in general, Rho GTPases are activated in membrane protrusions to promote persistent migration in part by regulating protrusion of the leading edge [36], [37]. Taken together, our data suggest that TEM4 promotes activation of RhoC in endothelial cells and is likely to signal through RhoC to promote persistent cellular migration.

### TEM4 and RhoC Regulate Actin Network Organization in Protruding Membranes

**Figure 4:**
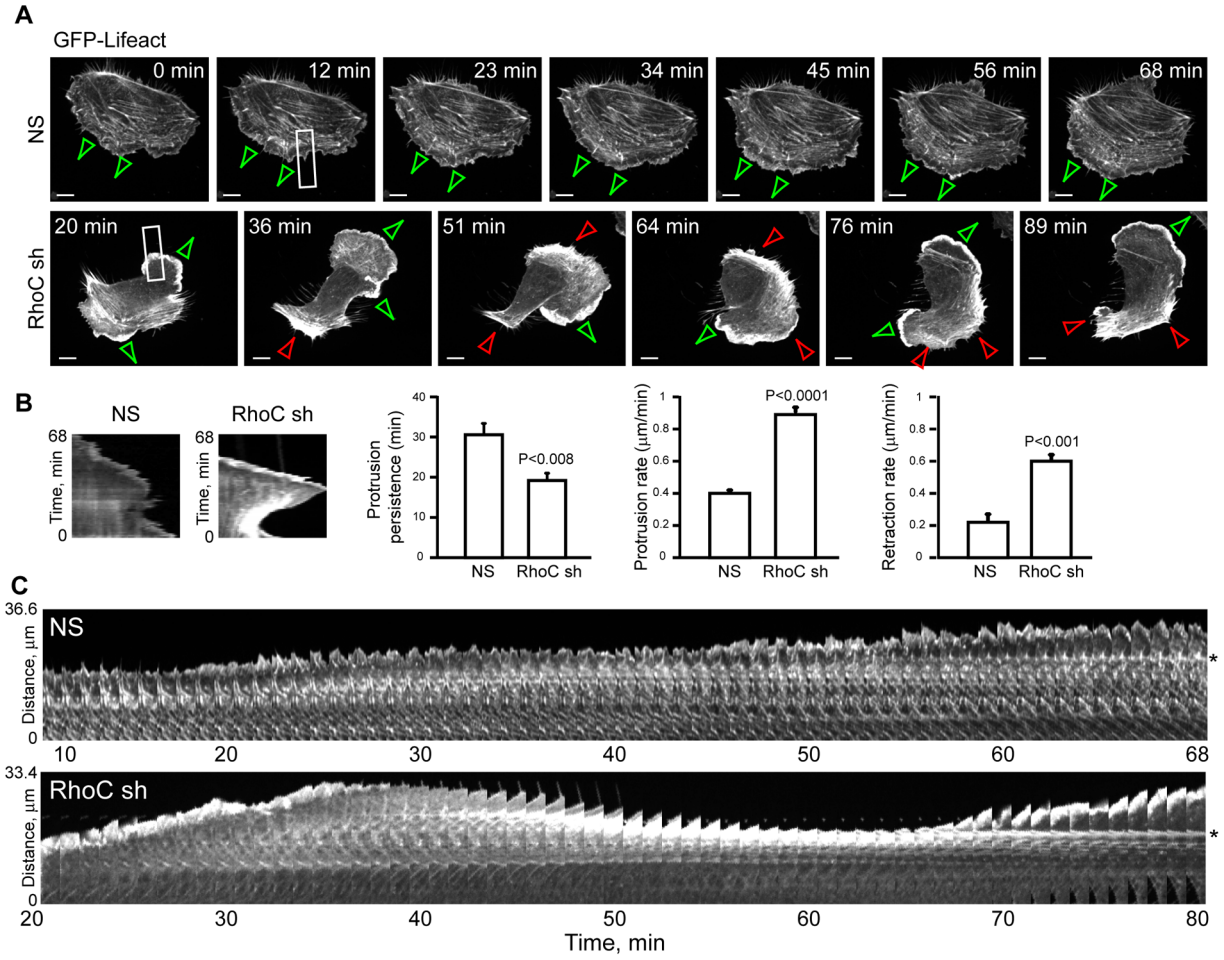
RhoC is required to maintain protrusion dynamics of the leading edge. (A) Frames of a time-lapse movie recording GFP-Lifeact to demonstrate organization of the actin filaments during migration of NS control (top row) or RhoC-depleted cells (bottom row). Green arrows indicate leading edge protrusion, with red arrows indicating edge retraction. Scale bar, 10 µm. (B) Kymography analysis of membrane protrusions of NS or RhoC-depleted cells. Sample kymographs and protrusion parameters of HUVECs depleted of RhoC or NS control. In each experimental group, 5–6 protrusions per cell in each of 4–6 cells were analyzed and data are mean ± s.e.m. (C) Time lapse montage of an area outlined in panel A showing a close up of actin filaments in protruding membranes of NS control (top row) or RhoC-depleted cells (bottom row). Asterisk marks an individual F-actin filament to highlight differential appearance between actin filaments in NS and RhoC-depleted cells.

Proper organization of the actin cytoskeleton at the leading edge of migrating cells is essential for membrane protrusion and persistent cell movement [2], [5], [6]. Given the essential role of Rho GTPases in regulating the polymerization and reorganization of the actin cytoskeleton, we hypothesized that TEM4 and RhoC regulate membrane protrusion and, therefore, cell migration by regulating the actin cytoskeleton in protruding membranes. To test our hypothesis, we examined the F-actin cytoskeleton in cells depleted of RhoC by time-lapse imaging of cells expressing F-actin marker, Lifeact. First, we observed membrane protrusions in whole cells. Time-lapse imaging of NS control cells expressing GFP-Lifeact revealed directional protrusion of the leading edge (Fig. 4 A top row, green arrowheads and Movie S6). In contrast, membrane protrusions in RhoC-depleted cells were prone to multiple rounds of retraction (Fig. 4 A bottom row, red arrowheads), each followed by membrane protrusions of the collapsed areas (Fig. 4 A bottom row, green arrowheads and Movie S7). Kymography analysis of RhoC- depleted cells revealed that protrusions of the leading edge had a much shorter persistence and altered protrusion/retraction rates of the leading edge as compared to NS control cells. This leading edge behavior led to frequent turning events observed in whole cell migration analyses (Fig. 2).

**Figure 5:**
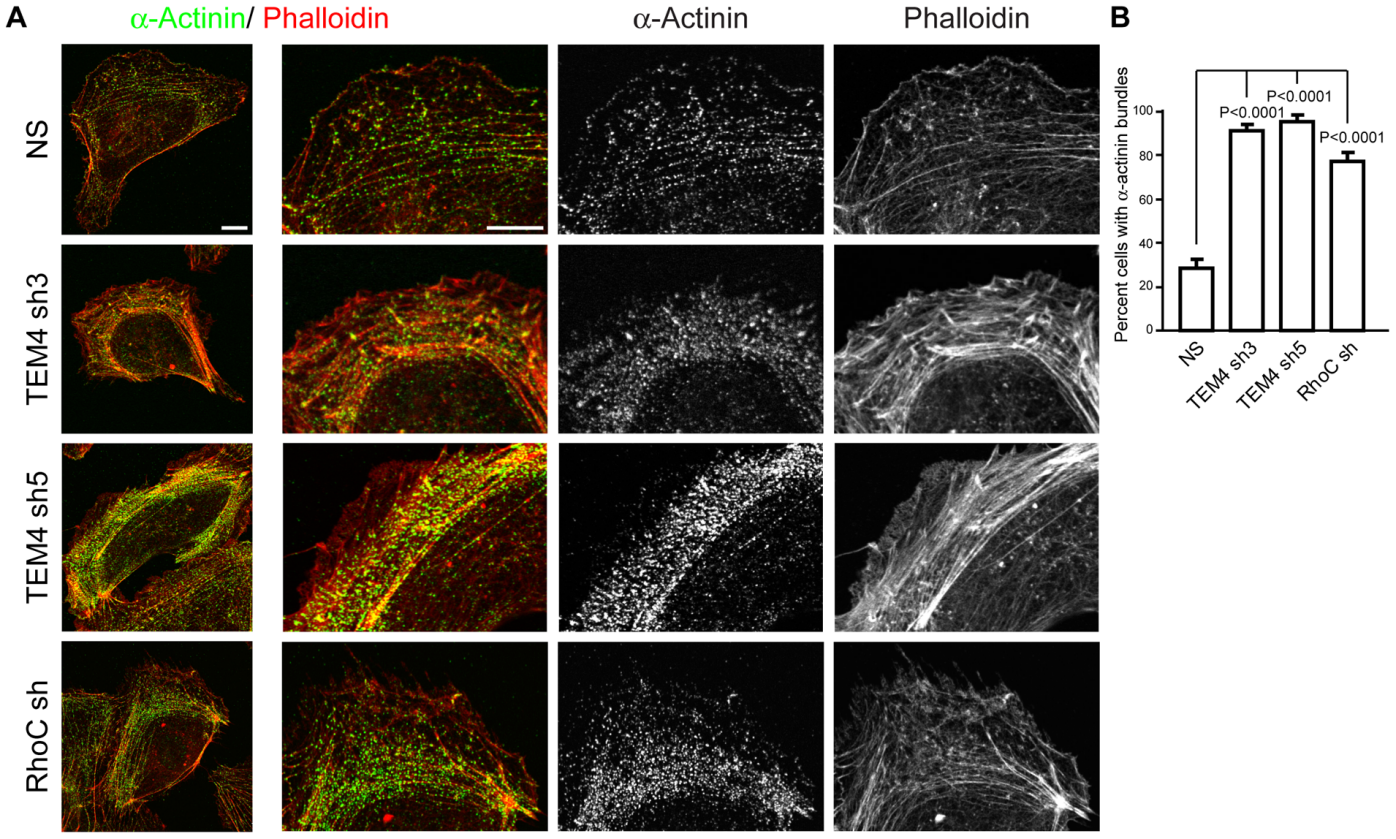
TEM4 and RhoC are required to maintain proper actin cytoskeleton architecture in lamella. (A) Actin filaments were visualized by staining with phalloidin and α-actinin 1 in NS control or cells depleted of TEM4 and RhoC. Cells were prepermeabilized with Triton X-100 to remove cytosolic α-actinin 1. The whole cell and an isolated area of the leading edge are shown. Scale bar, 10 µm. (B) Depletion of TEM4 or RhoC increases the number of cells with tight actin/α-actinin bundles. Data are mean ± s.e.m. obtained from 42–57 cells in three independent experiments.

Second, we inspected the actin cytoskeleton in protruding membranes of the leading edge. As shown in Figure 4 C, F-actin filaments appeared dramatically different between control and RhoC-depleted cells. In NS control cells, individual well-spaced actin filaments that ran parallel to the protruding edge were easily identifiable (Fig. 4 C top row). In contrast, in RhoC-depleted cells, actin stress fibers remained parallel to the protruding edge but were present in thick bundles (Fig. 4 C bottom row and data not shown). These bundles formed during strong retractions of the leading edge as the existing individual actin filaments collapsed onto one another (Fig. 4 C bottom row, asterisk). These thick actin stress fibers persisted even as the membrane began to protrude again (Fig. 4 A bottom row, 76 and 89 min time frame) and were observed in a large majority (80–100%) of TEM4- or RhoC-depleted cells (Figure 5 A–B). Our data suggested that TEM4 and RhoC were essential for the regulation of actin cytoskeleton in protruding membranes. The abnormal actin stress fibers in TEM4- or RhoC-depleted cells could be an underlying cause for the membrane protrusion defects and would account for the loss of persistence in whole cell migration assay.

### TEM4 and RhoC Suppress Myosin Contractility

**Figure 6:**
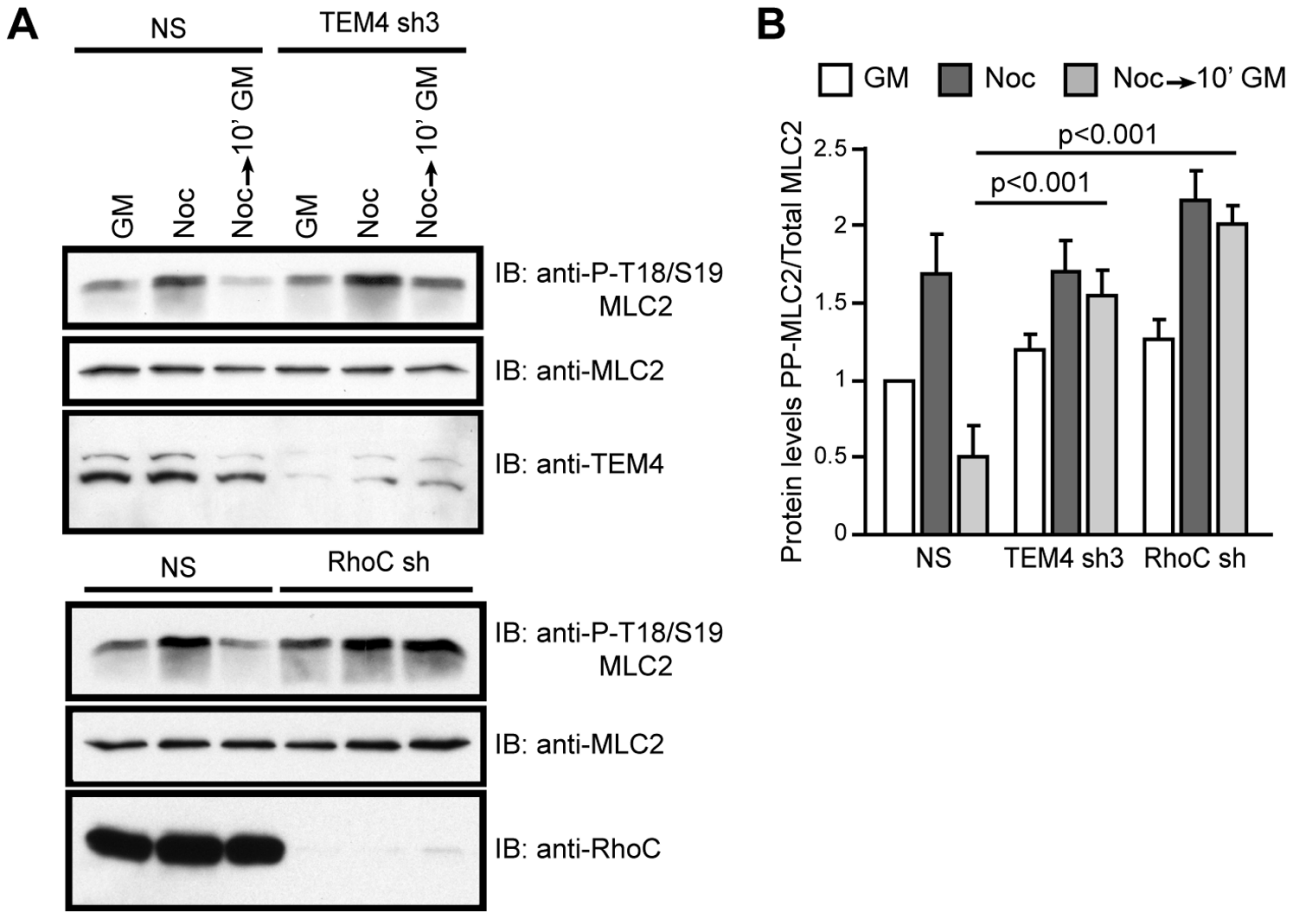
TEM4 and RhoC are required to suppress myosin contractility. Knockdown of TEM4 or RhoC impairs cellular ability to down regulate myosin contractility. Cells depleted of TEM4 or RhoC or NS control were left untreated (GM), treated with nocodazole (Noc) or treated with nocodazole with subsequent nocodazole washout. Phosphorylation of MLC2 was determined by western blot analysis of whole cell lysates (A), quantitated using densitometry and graphed (B). Phospho-MLC2 levels in each group were normalized to levels in the untreated NS control cells that were set to 1. Data are mean ± s.e.m. measured in three independent experiments.

Actomyosin contractility is an essential regulator of actin cytoskeleton and promotes maintenance of actin filaments in protruding membranes [5], [6]. Given its critical role in maintaining the actin cytoskeleton, actomyosin contractility needs to be tightly controlled during membrane protrusion/retraction phases to allow for efficient cell migration. Therefore, we suspected that excessive actomyosin contractility in cells depleted of TEM4 or RhoC may be an underlying cause of protrusive area collapse and the appearance of abnormal actin filaments. To determine if actomyosin activity was elevated in cells depleted of TEM4 or RhoC, we measured the phosphorylation of the regulatory myosin light chain (MLC2), which is an indicator of myosin II activity. We did not observe a significant overall difference in the levels of phospho-MLC2 in cells maintained under steady-state conditions in growth media (Fig. 6, GM). Therefore, we acutely stimulated myosin contractility by treatment with nocodazole and allowed contractility to return to a basal level during nocodazole washout [38], [39], [40]. Nocodazole treatment caused a significant increase in the level of phospho-MLC2 (Fig. 6) that was not altered by the depletion of TEM4 or RhoC (Fig. 6). However, when compared to control NS cells, where a rapid decline of phospho-MLC2 levels following the nocodazole washout was seen, depletion of TEM4 or RhoC prevented this decline, leading to persistence in phospho-MLC2 levels (Fig. 6; 10 min GM). Therefore, we concluded that TEM4 and RhoC act to restrict myosin II activity to allow protrusion of the leading edge and persistent cellular migration.

The finding that TEM4 suppresses contractility was somewhat surprising as TEM4 possesses catalytic activity towards RhoA [15], a well-known activator of actomyosin contractility [41], [42], [43]. Therefore, we measured activation of RhoA in cells depleted of TEM4 or RhoC under nocodazole washout conditions used to measure contractility changes. As shown in Figure S4, RhoA was hyperactivated in cells depleted of TEM4 or RhoC suggesting that TEM4 and RhoC antagonize contractility by antagonizing the activation of RhoA. It should be noted that the observed increase in RhoA total protein levels in RhoC-depleted cells is consistent with a previous study [44] and a result of stabilization of RhoA protein by a RhoGDI. Although the mechanism of selective engagement of RhoC over RhoA by TEM4 remains to be discovered, the ability of cells to antagonize RhoA activation at the leading edge is essential for membrane protrusion and cellular migration [45], [46], [47], [48], [49].

### TEM4 and RhoC Control Focal Adhesions

**Figure 7:**
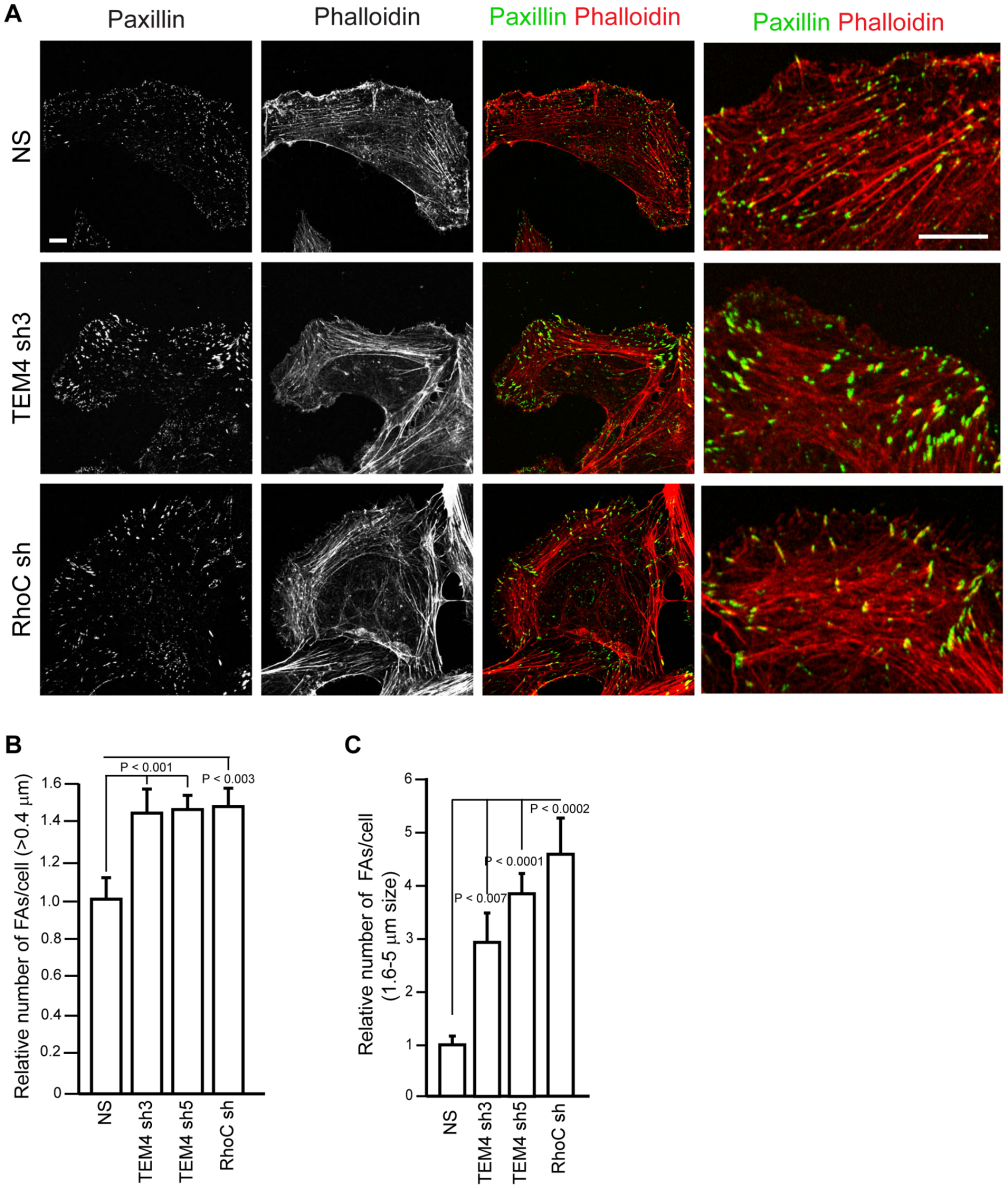
Depletion of TEM4 and RhoC causes accumulation of enlarged focal adhesions. (A) Focal adhesions in NS control, TEM4- and RhoC-depleted HUVECs were visualized by staining with paxillin and phalloidin. The whole cell and an isolated area of the leading edge are shown. Scale bar, 10 µm. (B, C) Depletion of TEM4 or RhoC leads to an increase in a total number of FAs (B) and number of large FAs (C). Data are mean ± s.e.m. measured in 10 cells in two independent experiments.

We next determined if TEM4 and RhoC regulate cell-substrate adhesion. Actin stress fibers in protruding membranes are coupled to focal adhesions (FAs) linking a cell to substrate to provide a traction network for leading edge advance and whole cell body translocation. Therefore, an increase in contractility (Fig. 6), as well as an increase in F-actin stress fibers (Fig. 5) in TEM4- and RhoC-depleted cells would promote a stronger cell-substrate attachment through FAs and thus impair effective membrane protrusion. To determine if TEM4 and RhoC regulate FAs, we depleted TEM4 or RhoC and calculated the number of FAs per cell by immunofluorescent detection of endogenous paxillin [50], [51], [52]. As expected, control cells displayed small (less than 0.4 µm [53], [54]) paxillin-positive structures indicative of focal complexes at the cell edge, and FAs that flanked each actin filament in protruding membranes (Fig. 7 A, B). However, although TEM4 or RhoC knockdown cells were still able to assemble focal complexes, there was a dramatic increase (∼ 40%) in the total number of FAs and a number of large FAs (Fig. 7 A–C). Therefore, we suggest that depletion of TEM4 or RhoC causes an increase in cell-substrate attachment which would be detrimental to efficient cellular migration.

### Suppression of ROCK Activity Rescues Persistent Migration of TEM4-depleted Cells

**Figure 8:**
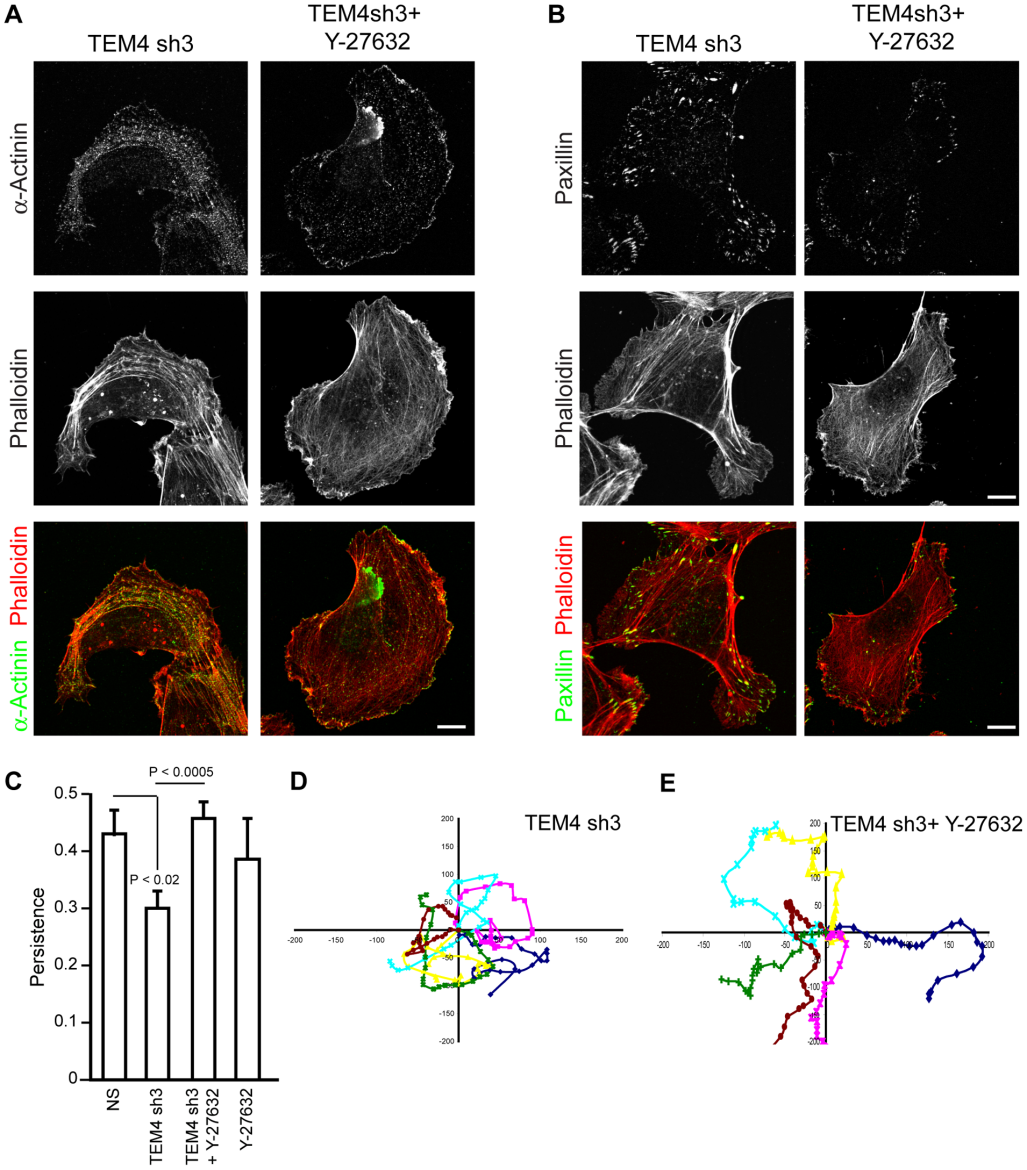
Suppression of ROCK activity in TEM4-depleted cells rescues persistence of cellular migration. (A) Inhibition of ROCK activity with Y-27632 normalized the appearance of actin cytoskeleton in TEM4-depleted cells. HUVECs were stained with phalloidin and antibody against α-actinin. (B) Enlarged FAs in TEM4-depleted cells disappear upon treatment with Y-27632. HUVECs were stained with paxillin antibody and phalloidin. Scale bar, 10 µm. (C) Persistence of 2D cellular migration of HUVECs expressing TEM4 shRNA is restored upon treatment with Y-27632. Data are mean ± s.e.m. measured from 17 (NS), 26 (TEM4 sh +/− Y-27632) and 14 (NS+Y-27632) individual cells in three independent experiments. (D, E) Wind-Rose plots depicting migratory tracks of six individual migrating cells in each experimental group. Values on x and y scales are arbitrary. Scale bar, 10 µm.

Our data suggest that TEM4 regulates the actin cytoskeleton and modulates cell-substrate adhesion to promote persistent cellular migration. In addition, TEM4 may function to suppress actomyosin contractility as cells with decreased levels of TEM4 expression had higher levels of active myosin. However, it is not clear if the ability of TEM4 to suppress myosin contractility is essential for its role in cellular migration. To assess if suppression of myosin contractility is essential for the role of TEM4 in cellular migration, we determined if suppression of Rho-associated kinase (ROCK) activity, a well-established upstream regulator of myosin contractility [41], [42], would rescue the persistence of cellular migration of TEM4-depleted cells. Consistent with this possibility, treatment with the ROCK-selective Y-27632 pharmacologic inhibitor [55] reversed the accumulation of abnormal actin stress fibers and FA enlargement observed in TEM4-depleted cells (Fig. 8 A, B) and, more importantly, restored persistence of cellular migration of HUVECs depleted of TEM4 (Fig. 8 C–E and Movie S8). Treatment with Y-27632 alone did not impair persistence of cellular migration (Movie S9). Therefore, we conclude that TEM4-mediated suppression of myosin contractility is critical for the regulation of the actin cytoskeleton, cell-substrate adhesion and cellular migration.

## Discussion

In this study, we determined whether TEM4, a RhoGEF implicated in cancer-associated angiogenesis, regulates cellular migration. We demonstrated that TEM4 regulates persistence of endothelial cell migration and regulates the actin cytoskeleton network and focal adhesions in membrane protrusions at the leading edge of migrating cells. We also found that TEM4 functions to suppress actomyosin contractility and that an ability to regulate actomyosin contractility is essential for TEM4 function in cellular migration.

We found that the RhoGEF TEM4 controls the persistence of migration of endothelial cells and TEM4-RhoC signaling functions to restrict myosin contractility in migrating cells. Furthermore, suppression of myosin contractility is essential for TEM4 function in migrating cells, as treatment of cells with ROCK inhibitor, an upstream regulator of myosin II, reverses TEM4-mediated phenotypes. Even though the mechanism by which TEM4-RhoC suppress myosin II activity remains to be identified, localized inhibition of contractile forces at the leading edge of cells was previously shown to be essential for cell migration [47], [49] and for endothelial cell sprouting during angiogenesis [56], [57]. It has also been suggested that tumors utilize a contractility-independent mode of migration [58], [59] and it is possible that this occurs via a TEM4-RhoC signaling pathway. Future studies will focus on an investigation of the molecular mechanisms that drive TEM4- and RhoC-dependent endothelial and tumor cell migration and invasion to regulate tumor progression.

Depletion of either TEM4 or RhoC inhibits persistence of cellular migration, and alters actin cytoskeleton and FA morphology, suggesting that RhoC activation by TEM4 may mediate TEM4 functions. Consistent with this, we found that RhoC activation in protruding membranes was severely impaired upon TEM4 depletion. Interestingly, endogenous TEM4 associates with both microtubular and actin cytoskeletons in protruding membranes (Fig. S5) suggesting that association with either or both structures may regulate spatial activation of RhoC by TEM4 as shown for other RhoGEF family [60], [61]. We have previously shown that TEM4 contains an actin-binding domain which is required for its ability to activate RhoC *in vivo* [15], whereas the mechanism of association of TEM4 with microtubules is unknown. Further studies are required to determine if the association of TEM4 with cytoskeletal components dictates spatial activation of RhoC and if this association is essential for TEM4 function in cell migration.

Our findings that TEM4-RhoC suppresses phosphorylation of MLC2 are unexpected. In general, Rho family proteins are thought to activate MLC2 by signaling through Rho-associated kinase (ROCK) [62], [63]. However, several studies suggested that RhoC functions independently of ROCK signaling [64], [65] and a recent study reported that RhoC suppresses MLC2 phosphorylation in response to LPA in osteosarcoma and ovarian cancer cells [66]. Given the essential role of RhoC in tumorigenesis [67], [68], [69], [70], [71], future studies are required to determine the mechanism by which RhoC mediates suppression of myosin II activity and, possibly, tumorigenesis.

The ability of TEM4 and RhoC to regulate leading edge protrusion undoubtedly stems from the regulation of actin stress fibers, formation and maintenance of which are essential for membrane protrusion [5], [6]. Loss of TEM4 or RhoC promoted the accumulation of thick actin bundles in protruding areas of the cell suggesting that TEM4-RhoC signaling regulates actin stress fiber formation and distribution. The mechanisms by which TEM4-RhoC regulate the actin cytoskeleton may involve fine tuning actomyosin contractility which is required for stress fiber formation and maintenance [4], [5]. In addition, failure to disassemble FAs may also promote the accumulation of actin stress fibers at the leading edge observed in TEM4- or RhoC-depleted cells, as stress fibers associate with FAs to maintain spacing and turnover [5]. Indeed, inhibition of ROCK activity rescued the over bundled appearance of the actin stress fibers and the loss of cellular persistence of TEM4-depleted cells.

Whereas suppression of myosin contractility by an unknown mechanism is undoubtedly important for TEM4 function in cellular migration, there may be additional mechanisms utilized by TEM4 to regulate lamellar actin architecture. One possibility is activation of other Rho effectors, such as formin proteins, by TEM4-RhoC signaling to regulate actin stress fiber polymerization. Loss of the formin mDia1 has been shown to impair actin polymerization and loss of actin stress fibers was observed in cells treated with a broad-specificity chemical inhibitor of formin [4], [72]. In addition to its role in actin polymerization, mDia1 has also been shown to promote FA disassembly at the leading edge by recruiting active Src kinase to FAs downstream of Rho GTPases [73] and could therefore promote the stabilization of actin stress fibers that we observed in cells depleted of TEM4 or RhoC. Recent studies identified additional members of the formin family as mediators of RhoC function [65], [74]; however, the mechanism by which these formins promote cellular migration and invasion remains unknown.

## Materials and Methods

### Expression Constructs and Antibodies

Lifeact-GFP constructs were made by subcloning sequences encoding the Lifeact [35] N-terminus to GFP in the pLL 5.0 lentiviral vector [75].Antibodies used in this study were as following: TEM4 (4367; ProSci Inc), α-tubulin (DM1A; Sigma), α-actinin (BM-75.2; Sigma), β-actin (AC-15; Sigma), anti-paxillin and anti-PECAM (MEC13.3) (BD Transduction), anti-RhoA, anti-RhoC, anti-MLC2 and anti-phospho MLC2 (Cell Signaling). Alexa 594-conjugated phalloidin and Alexa-conjugated secondary antibodies for the immunocytochemistry were from Invitrogen. Nocodazole (Sigma) and Y-27632 (Calbiochem) were used at final concentration of 10 µg/ml and 10 µM, respectively.

### Endothelial Cell Culture, Lentivirus Production and Transduction

293T cells (ATCC) were maintained in high glucose Dulbecco’s modified Eagle’s medium (DMEM) supplemented with 10% fetal bovine serum (FBS). For virus production, 293T cells were transfected using a standard calcium phosphate DNA precipitation method with a target vector and the ViraPower lentiviral packaging system (Invitrogen). Supernatant containing the virus was collected 48 h post transfection and the titer was determined by infecting fresh 293T cells.

Human umbilical vein endothelial cells (Lonza) were maintained in EGM-2 medium supplemented with 10% FBS (HyClone) and endothelial cell culture additives on gelatin-coated dishes (0.01%). For lentiviral transduction, HUVECs were incubated with the virus (MOI<10) for 4–5 h in EGM-2 medium in the absence of serum. HUVECs were passaged 24 h after transduction and used for experiments 24–48 h later. All experiments were carried out in HUVECs between passages four and five.

### ES Cell Culture and in vitro Differentiation

ES cells [22] were grown in ES medium (66% of 5637 conditioned medium [76], 17% FBS, 82.5 µM monothioglycerol (MTG), 15% DMEM, and 0.5 µg/ml gentamicin) in dishes coated with 0.1% gelatin. For lentiviral transduction, ES cell cultures were incubated with undiluted viral supernatant for 4–5 h and allowed to recover in ES cell medium overnight. 72h later, ES cells were passaged and infected again using the same infection protocol. Infection efficiency was estimated 48 h after the second infection at 50–70%.

ES cell cultures were differentiated as described previously [22], [77]. Briefly, ES cells were detached (day 0) with 2.4 U/ml dispase (Grade II stock, Boehringer-Mannheim) and replated in bacteriologic dishes in differentiation media (DMEM supplemented with 20% FBS and 150 µM MTG). EBs were transferred to a plate with fresh differentiation media on day 2. The next day (day 3), EBs were transferred into gelatin-coated tissue culture plates in differentiation media and fed every other day until day 8, when the cultures were fixed and analyzed. For RNA isolation, EBs were plated into 100 mm (earlier time points) and 60 mm plates (later time points). For immunocytochemistry, EBs were plated in 24 well plates.

### Antibody Staining and Image Analysis of ES Differentiation Assay

Day 8 ES cell cultures were fixed with 4% PFA in PBS for 5 min and stained with anti–PECAM antibody. PECAM-stained cultures were viewed and photographed with an inverted microscope (IX-50; Olympus) outfitted with epifluorescence using a 10×NA 0.25 CPlan RT objective (Olympus) and a camera (DP71; Olympus) with the DP Controller version 3.1.1.267 software (Olympus). Minor adjustments (brightness and contrast to the whole panel) were performed using Photoshop CS3 (Adobe). To quantify the vascular area labeled with PECAM antibody, four wells were analyzed for each test group. For each well, six sequential non-overlapping areas completely covered with cells were photographed at 10x magnification, so that the total area photographed per well was more than 60% of the well. Percent PECAM area was calculated for each image by adjusting threshold so the entire vessel is filled and measuring vessel area using ImageJ “Analyze Particles” tool. Means for each well were calculated, and the mean of four wells for each test group was used for statistical analysis.

### Matrigel in vitro Angiogenesis Assay

Wells of 12-well plates were coated with growth-factor reduced Matrigel (9 mg/ml; BD BioSciences). HUVECs expressing NS control or TEM4 shRNA were resuspended in EGM-2 and seeded on top of solidified Matrigel matrix (6×10^4^ cells/well) in triplicate. Tubule formation was monitored for 24 h where cells were photographed at various time points. For each well, 4 fields/well were photographed for each time-point and number of enclosed spaces was counted manually and averaged.

### RT-PCR Analysis

RNA was isolated using the RNeasy Mini Kit (Qiagen) according to the manufacturer’s protocol. iScript cDNA synthesis kit (Bio-Rad) was used for reverse transcription and cDNA was amplified using Taq polymerase for a number of cycles were linear amplification was observed. Primers used for the PCR- Tem4 forward primer, 5′- CTGGAGGACCATGAGCAGT -3′; reverse primer, 5′- GGCTGATGGCTTTTTGGAT-3′; VEGFR-1/Flt-1 forward primer, 5′- TGTGGTCCTATGGCGTGTT-3′; reverse primer, 5′- ATCTTCATGGAGGCCTTGG-3′; Gapdh forward primer, 5′- AACTTTGGCATTGTGGAAGG -3′; reverse primer, 5′- TGTGAGGGAGATGCTCAGTG -3′). In each case, primers were designed to flank an intron and no amplification of genomic DNA was detected.

### RNA Interference

Short hairpin (shRNA) oligos were subcloned into pLL 5.0 GFP [75] or pLL 5.0 mCherry lentivirus vectors or pLL5.0 vector where fluorescent marker has been deleted. Target sequences were as following: TEM4 #3 5′-GCACCACTCTGAAGCGAA-3′; TEM4 #5 5′-GGAAATGACATGAGGAAA-3′; RhoC 5′-GGATCAGTGCCTTTGGCT -3′; RhoA 5′-GGAAGAAACTGGTGATTG-3′; and mouse TEM4 5′- GAACAAGGACTATCAGGAA -3′. The control shRNA (NS; GATCGACTTACGACGTTAT) has no exact match in the human, mouse or rat genomes [75].

### Single Cell Tracking and Kymography Analysis

HUVECs were sparsely plated on a gelatin-coated MatTek dish in growth medium and allowed to attach overnight. 20×0.5 NA phase time-lapse movies were recorded for 2.5 h with frames taken every 5 min on a Nikon Biostation IM (Nikon) equipped with DS-2MBWC camera (Nikon). In each experiment 10–12 fields were simultaneously recorded using an automated stage. For the analysis, every cell in each movie that met the tracking criteria (was completely within the field of view for the entire experiment, did not divide, and did not touch another cell for more than three frames) was tracked using MTrackJ plug-in for ImageJ with the point-click mode. In each case, cell centroid, defined as a half point along the long axis of the cell, was used for tracking. Data were exported into Microsoft Excel for analysis. The persistence of migration was calculated as [net displacement from origin (µm)/total length of migration paths (µm)].

For GFP-LifeAct kymography, videos were recorded for 1–2 h with frames taken every 60 sec on an Axio Observer microscope (Zeiss) with a plan-apochromat 63×1.4 NA objective. Kymographs were assembled from equal-length videos for each individual cell using ImageJ and lamellipodial parameters were calculated as described [75]. Five to six lines were drawn perpendicular to the protrusive areas in each of 4 to 6 cells and lamellipodial parameters were calculated using custom script [75]. Parameters for each individual protrusive area within a cell were averaged and means of 4–6 cells were used for statistical analysis.

### Bimolecular Fluorescent Complementation Analysis of Activation of RhoC

For experiments determining the specificity of BiFC reagents, 293T cells were plated in 6-well plates and transfected with VN-RhoC, VC-RBD and tRFP (200 ng each plasmid) using calcium phosphate precipitation method. Eighteen h after transfection the cultures were trypsinized and replated into glass-bottom MatTek dishes in complete growth medium. The fluorescence derived from the BiFC and tRFP markers was visualized 24 h after transfection using spinning disk confocal microscope (Axio Observer; Zeiss) with a plan-apochromat 63×1.4 NA objective. Images were captured by sequential scanning with the 488 nM argon and the 561 nM HeNe1 laser and the BP 525/50 (for BiFC), BP 598 (for tRFP) emission filters. To remove bias, tRFP-positive cells were identified without checking for presence of the BiFC signal and fluorescent intensities produced by BiFC and the tRFP internal reference were recorded for each cell. To process images, cell boundary was identified using tRFP channel and total cell fluorescence intensity was measured in ImageJ and background subtracted. Finally, the ratio of BiFC to tRFP fluorescence intensity was obtained and plotted.

To analyze dynamics of activation of RhoC in HUVEC, cells were first infected with lentivirus encoding Lifeact-tRFP and TEM4 shRNA #3 or NS control. In this instance, mCherry fluorescent marker was deleted from TEM4 shRNA pLL 5.0 vector by restriction digestion. Twenty-four h after the first infection, cells were infected with lentivirus encoding VN and VC fusions. Cells were subsequently split into glass-bottom MatTek dishes and visualized 72 h after the first infection using a Zeiss Observer and 63×1.4 NA objective in microscopy medium (DMEM/F12 with endothelial growth medium bullet kit (Lonza) supplemented with 10% FBS (Characterized FBS; HyClone)). Time-lapse images were collected every min using the following exposure times: 550 ms for tRFP and 500 ms for BiFC-RhoC. Image brightness and contrast were adjusted in ImageJ software. To calculate fluorescence intensity of RhoC-BiFC, a 10 µm-wide rectangle was drawn at the protrusive edge or inside the cell (forward of the nucleus) and intensity in each frame of the time lapse was measured using plot Z-axis profile tool in ImageJ. These measurements were used to calculated edge/cell body ratio fluorescence.

### Immunocytochemistry and Microscopy

HUVECs were fixed with 4% paraformaldehyde in cytoskeletal buffer (CS; 5 mM PIPES, pH 6, 137 mM NaCl, 5 mM KCl, 1.1 mM Na_2_HPO_4_, 0.4 mM KH_2_PO_4_, 0.4 mM MgCl_2_, 0.4 mM NaHCO_3_, 2 mM EGTA, 50 mM glucose) for 15 min. In experiments described in Figs. 5 and 8, cytosolic α-actinin was eluted by a simultaneous fixation-prepermeabilization protocol (5 min in 0.5% Triton X-100, 3% PFA in CS buffer, 15 min in 3% PFA in CS). Cells were permeabilized with 0.2% Triton X-100 for 5 min, blocked with 5% BSA and stained with antibodies. FluorSave reagent (EMD Chemicals) was used as the mounting media. Cells were examined with an inverted laser scanning confocal microscope LSM 510 (Carl Zeiss, Inc) using an oil immersion plan-apochromat 63×1.4 NA objective. Images were captured by sequential scanning with the 488 nM argon/633 nM HeNe2 lasers and the 543 nM HeNe1 laser (488 nM and 543 nM for 2-color staining), and the BP 505–530 (for Alexa 488), BP 585–615 (for Alexa 594 and mCherry), or LP 650 (for Alexa 647) emission filters and recorded by the LSM software (Zeiss). For scoring, images were captured using plan-apochromat 40×1.2 NA objective and number of cells with tight bundles of actin crosslinked by α-actinin was counted manually. Image brightness and contrast were adjusted using Adobe Photoshop CS3.

### Focal Adhesion Analysis

Cells were plated on gelatin-coated 15-mm glass slides and allowed to attach overnight. Cells were fixed using a simultaneous permeabilization-fixation protocol and stained with rabbit anti-paxillin antibodies and phalloidin. Only cells with a single nucleus were used for the analysis to avoid variations in a total number of FAs due to cell size. The number of FAs per cell was quantitated from thresholded images using ImageJ. Raw time-lapse data were used for measurement of FA dynamics. For presentation purposes, brightness and contrast were adjusted using Adobe Photoshop CS3.

### Statistical Analysis

Prizm software was used for statistical analysis. In each case, p-values were calculated using a two-tailed unpaired *t*-test assuming unequal variance. Significance for all tests was assumed at p<0.05 (alpha 0.05).

## Supporting Information

Figure S1 **Lentiviral vector-based shRNA plasmid design used in the study.** (A) Diagram of the modified lentiviral vector combining shRNA expression from the Pol III U6 promoter with GFP or mCherry fluorescent proteins expression from the MSCV 5′ LTR promoter to identify infected cells for the analysis. (B) HUVECs were infected with TEM4 shRNA #3 co-expressing mCherry. Twenty-four h after infection cells were fixed and stained with nuclear marker. Infection efficiency was determined by counting cells not expressing mCherry (uninfected cells; marked with an arrowhead) and is 96% for the field shown. At the chosen MOI (<10) lentiviral infection efficiency neared 100% in every experiment.
(TIF)

Figure S2 **Characterization of BiFC-RhoC probe specificity.** (A) Schematic diagram of lentiviral vector-based shRNA plasmids used in the study. ORFs are drawn not to scale. (B-D) The wild-type or mutated RhoC and RBD were co-transfected into 293T with an internal control (tRFP). (B) Western blot confirming the expression of BiFC constructs in 293T cells. GFP antibody was used to detect VN fusions. Antibody against ROCKI was used to detect VC fusions. (C-D) Fluorescent intensities produced by BiFC and the internal reference were measured in individual cells (30–54 cells for each group) and the ratio is displayed as a distribution between individual cells (D) or a mean (C).
(TIF)

Figure S3 **BiFC-RhoC in endothelial cells.** (A) Individual frames from time-lapse movies show the fluorescent levels of RhoC-BiFC, GFP-RhoC or GFP alone in HUVECs. Arrows mark protruding areas of the cell. (B) Western blot analysis of BiFC constructs in HUVECs. RhoC antibodies were used to compare expression levels of VN-RhoC fusions to the endogenous RhoC. Antibody against ROCKI was used to detect the VC fusions. (C) BiFC requires wild type RhoC and ROCK as mutation of either RhoC or ROCK abolished BiFC-derived signal. (D–E) Activation of RhoC in NS control (D) or TEM4-depleted cells (E). BiFC-RhoC fluorescent signal was recorded in four cells in each experimental group. Scale bar, 10 µm.
(TIF)

Figure S4 **TEM4 and RhoC antagonize activation of RhoA.** (A) Knockdown of TEM4 or RhoC promotes activation of RhoA. Cells depleted of TEM4 or RhoC or NS control were left untreated (GM), treated with nocodazole (Noc) or treated with nocodazole with subsequent nocodazole washout. Active RhoA was pulled down in GTPase pull-down assay and levels of active and total RhoA were determined by western blot analysis. (B) Western blot confirming knockdown of RhoA and RhoC expression levels by lentivirus-based RNAi constructs in cells used for single cell tracking. NS; non-specific shRNA. (C) Persistence of two-dimensional cellular migration of HUVECs expressing NS, RhoC or RhoA shRNAs or treated with Y-27632. (D) Wind-Rose plots depicting migratory tracks of six individual migrating cells in each experimental group. Values on x and y scales are arbitrary.
(TIF)

Figure S5 **Localization of endogenous TEM4 to actin filaments and microtubules in protrusive areas of the cell.** (A) HUVECs were stained with antibodies against TEM4 and α-tubulin and Alexa-594 phalloidin. The close-up of cell periphery (B) or cell body (C) is shown. Specificity of TEM4 staining was confirmed by preincubating the TEM4 antibody with TEM4 immunizing peptide (E) or control peptide (D) of similar length. Scale bar 10 µm.
(TIF)

Movie S1 **Migration of NS control HUVECs.** HUVECs were infected with lentivirus encoding NS control shRNA and plated on gelatin-coated plates. Cellular migration was recorded using a bright field microscope (Nikon Biostation IM) for 2.5 h with an acquisition rate of 5 min/frame. Movies played at a speed of 5 frames-per-second. Scale, 10 µm.
(MOV)

Movie S2 **Migration of HUVECs with decreased expression of TEM4.** HUVECs were infected with lentivirus encoding TEM4 shRNA #3 and plated on gelatin-coated plates. Cellular migration was recorded using a bright field microscope (Nikon Biostation IM) for 2.5 h with an acquisition rate of 5 min/frame. Movies played at a speed of 5 frames-per-second. Scale, 10 µm.
(MOV)

Movie S3 **Migration of HUVECs with decreased expression of RhoC.** HUVECs were infected with lentivirus encoding RhoC shRNA and plated on gelatin-coated plates. Cellular migration was recorded using a bright field microscope (Nikon Biostation IM) for 2.5 h with an acquisition rate of 5 min/frame. Movies played at a speed of 5 frames-per-second. Scale, 10 µm.
(MOV)

Movie S4 **RhoC activation in NS control HUVECs.** HUVECs were infected with lentiviruses encoding Lifeact-tRFP and NS control. Twenty-four h after the first infection, cells were infected with RhoC-BiFC biosensor components. Time-lapse images of RhoC-BiFC (left panel) and Lifeact-tRFP (right panel) were recorded using a spinning disk confocal microscope (Zeiss Observer; Carl Zeiss, Inc.). Frames were recorded for 34 min with an acquisition rate of 1 frame/min and played at a speed of 5 frames-per-second. Scale bar, 10 µm.
(MOV)

Movie S5 **RhoC activation in TEM4-depleted HUVECs.** HUVECs were infected with lentiviruses encoding Lifeact-tRFP and TEM4 shRNA #3. Twenty-four h after the first infection, cells were infected with RhoC-BiFC biosensor components. Time-lapse images of RhoC-BiFC (left panel) and Lifeact-tRFP (right panel) were recorded using a spinning disk confocal microscope (Zeiss Observer; Carl Zeiss, Inc.). Frames were recorded for a total period of 41 min, using an acquisition rate of 1 frame/min and played at a speed of 5 frames-per-second. Scale bar, 10 µm.
(MOV)

Movie S6 **Actin cytoskeleton dynamics in control HUVECs.** HUVECs were infected with lentiviruses encoding NS control shRNA and Lifeact-tRFP. Time-lapse images of Lifeact-tRFP were recorded using a spinning disk confocal microscope (Zeiss Observer; Carl Zeiss, Inc.). Frames were recorded for 68 min with an acquisition rate of 1 frame/min and played at a speed of 5 frames-per-second. Scale bar, 10 µm.
(MOV)

Movie S7 **Actin cytoskeleton dynamics in RhoC-depleted HUVECs.** HUVECs were infected with lentiviruses encoding RhoC shRNA and Lifeact-tRFP. Time-lapse images of Lifeact-tRFP were recorded using a spinning disk confocal microscope (Zeiss Observer; Carl Zeiss, Inc.). Frames were recorded for 120 min with an acquisition rate of 1 frame/min and played at a speed of 5 frames-per-second. Scale bar, 10 µm.
(MOV)

Movie S8 **Treatment with Y-27632 restores persistence of HUVECs depleted of TEM4.** HUVECs were infected with lentivirus encoding TEM4 sh#3 shRNA and plated on gelatin-coated plates. Cells were pretreated with 10 µM Y-27632 for 30 min and cellular migration was recorded using a bright field microscope (Nikon Biostation IM) for 3 h with an acquisition rate of 5 min/frame. Movies played at a speed of 5 frames-per-second. Scale, 10 µm.
(MOV)

Movie S9 **Migration of control HUVECs treated with Y-27632.** Cells were pretreated with 10 µM Y-27632 for 30 min and cellular migration was recorded using a bright field microscope (Nikon Biostation IM) for 3 h with an acquisition rate of 5 min/frame. Movies played at a speed of 5 frames-per-second. Scale, 10 µm.
(MOV)

Results S1 (DOCX)
