# Supplementary figures and images for: The RhoGEF TEM4 Regulates Endothelial Cell Migration by Suppressing Actomyosin Contractility
Source: PLoS One. 2013 Jun 18;8(6):e66260. doi: 10.1371/journal.pone.0066260 (PMC3688894; doi:10.1371/journal.pone.0066260)

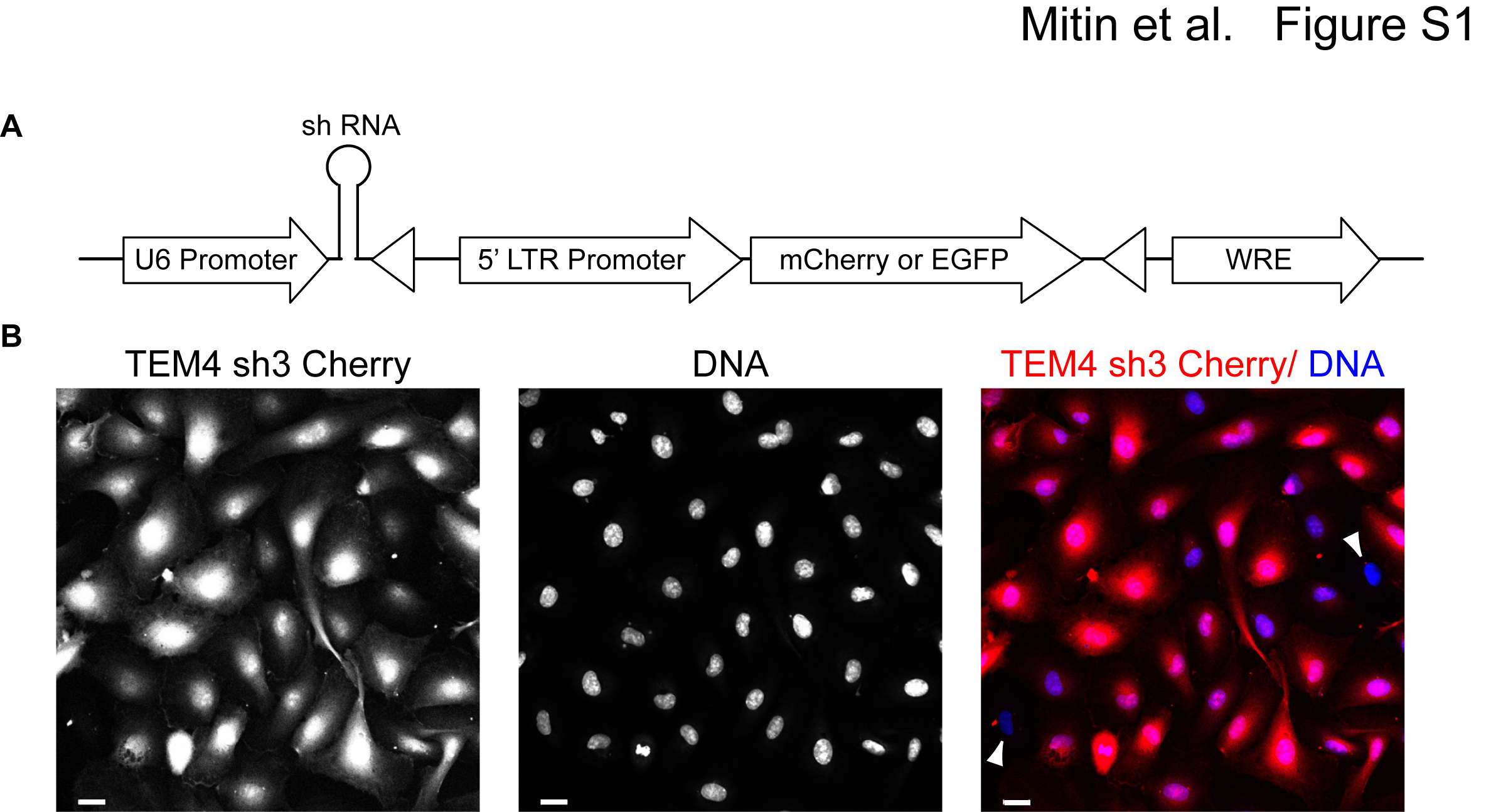

Supplement: Figure S1 — Lentiviral vector-based shRNA plasmid design used in the study. (A) Diagram of the modified lentiviral vector combining shRNA expression from the Pol III U6 promoter with GFP or mCherry fluorescent proteins expression from the MSCV 5′ LTR promoter to identify infected cells for the analysis. (B) HUVECs were infected with TEM4 shRNA #3 co-expressing mCherry. Twenty-four h after infection cells were fixed and stained with nuclear marker. Infection efficiency was determined by counting cells not expressing mCherry (uninfected cells; marked with an arrowhead) and is 96% for the field shown. At the chosen MOI (<10) lentiviral infection efficiency neared 100% in every experiment. (TIF) [file pone.0066260.s001.tif]

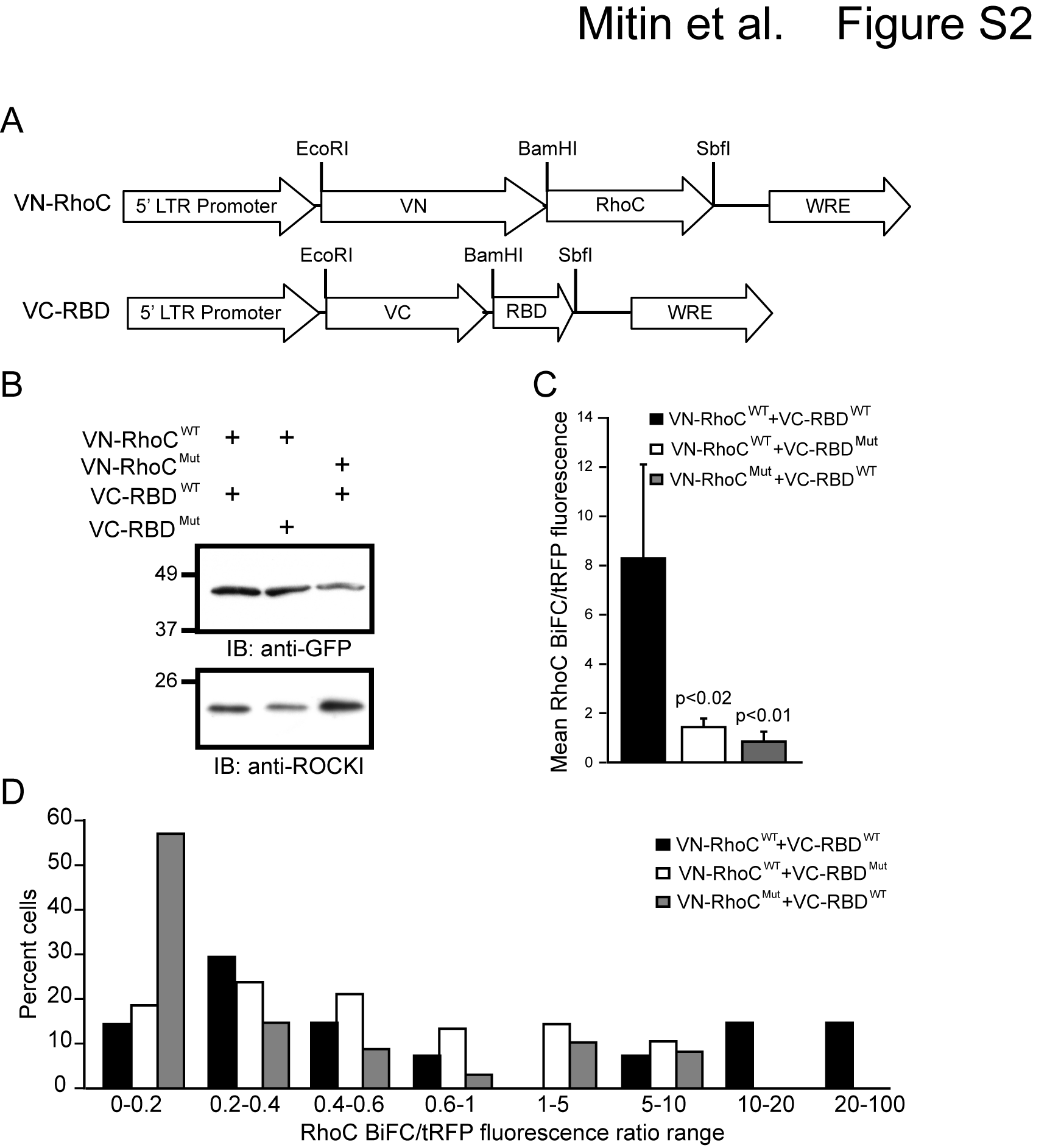

Supplement: Figure S2 — Characterization of BiFC-RhoC probe specificity. (A) Schematic diagram of lentiviral vector-based shRNA plasmids used in the study. ORFs are drawn not to scale. (B-D) The wild-type or mutated RhoC and RBD were co-transfected into 293T with an internal control (tRFP). (B) Western blot confirming the expression of BiFC constructs in 293T cells. GFP antibody was used to detect VN fusions. Antibody against ROCKI was used to detect VC fusions. (C-D) Fluorescent intensities produced by BiFC and the internal reference were measured in individual cells (30–54 cells for each group) and the ratio is displayed as a distribution between individual cells (D) or a mean (C). (TIF) [file pone.0066260.s002.tif]

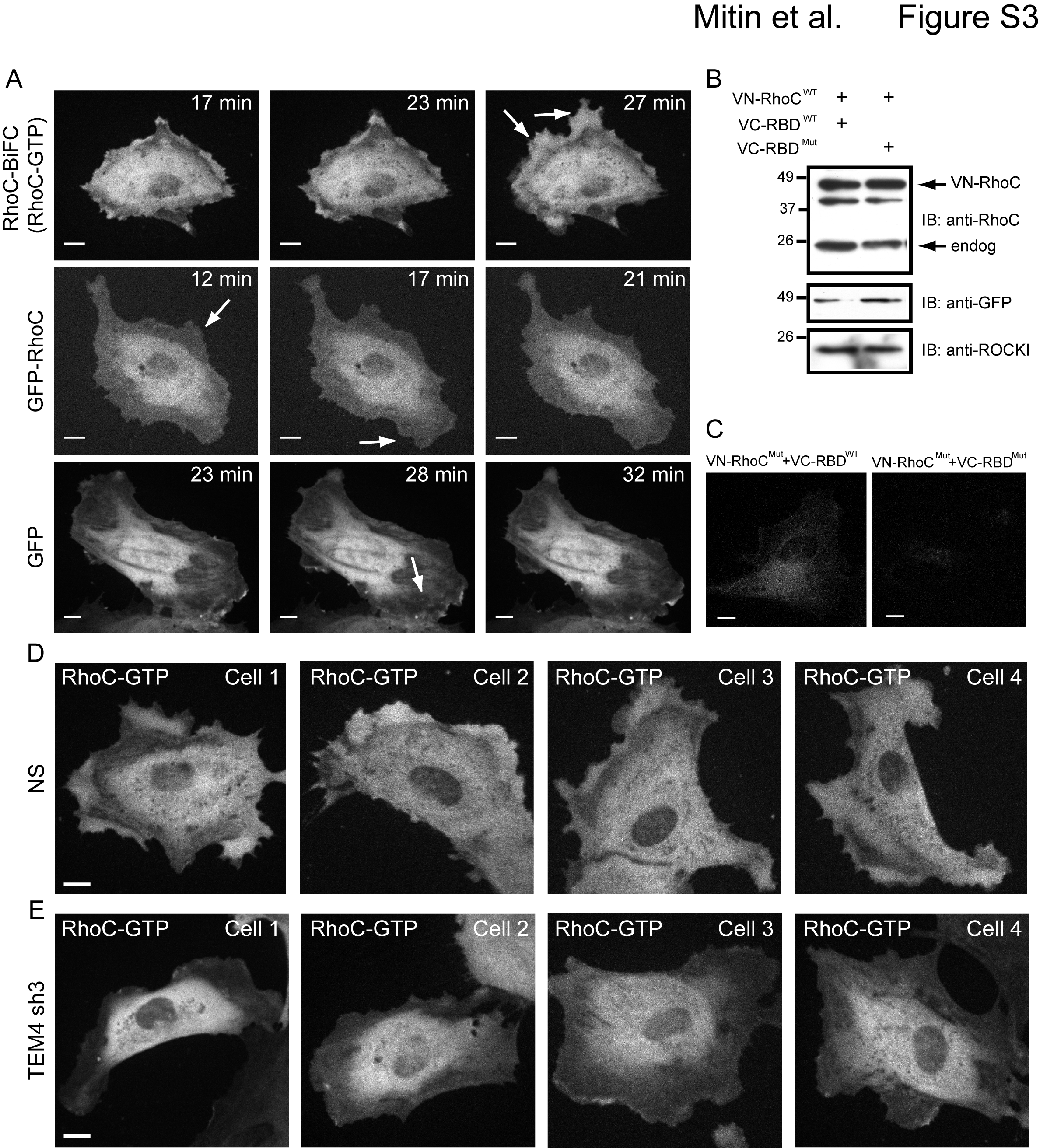

Supplement: Figure S3 — BiFC-RhoC in endothelial cells. (A) Individual frames from time-lapse movies show the fluorescent levels of RhoC-BiFC, GFP-RhoC or GFP alone in HUVECs. Arrows mark protruding areas of the cell. (B) Western blot analysis of BiFC constructs in HUVECs. RhoC antibodies were used to compare expression levels of VN-RhoC fusions to the endogenous RhoC. Antibody against ROCKI was used to detect the VC fusions. (C) BiFC requires wild type RhoC and ROCK as mutation of either RhoC or ROCK abolished BiFC-derived signal. (D–E) Activation of RhoC in NS control (D) or TEM4-depleted cells (E). BiFC-RhoC fluorescent signal was recorded in four cells in each experimental group. Scale bar, 10 µm. (TIF) [file pone.0066260.s003.tif]

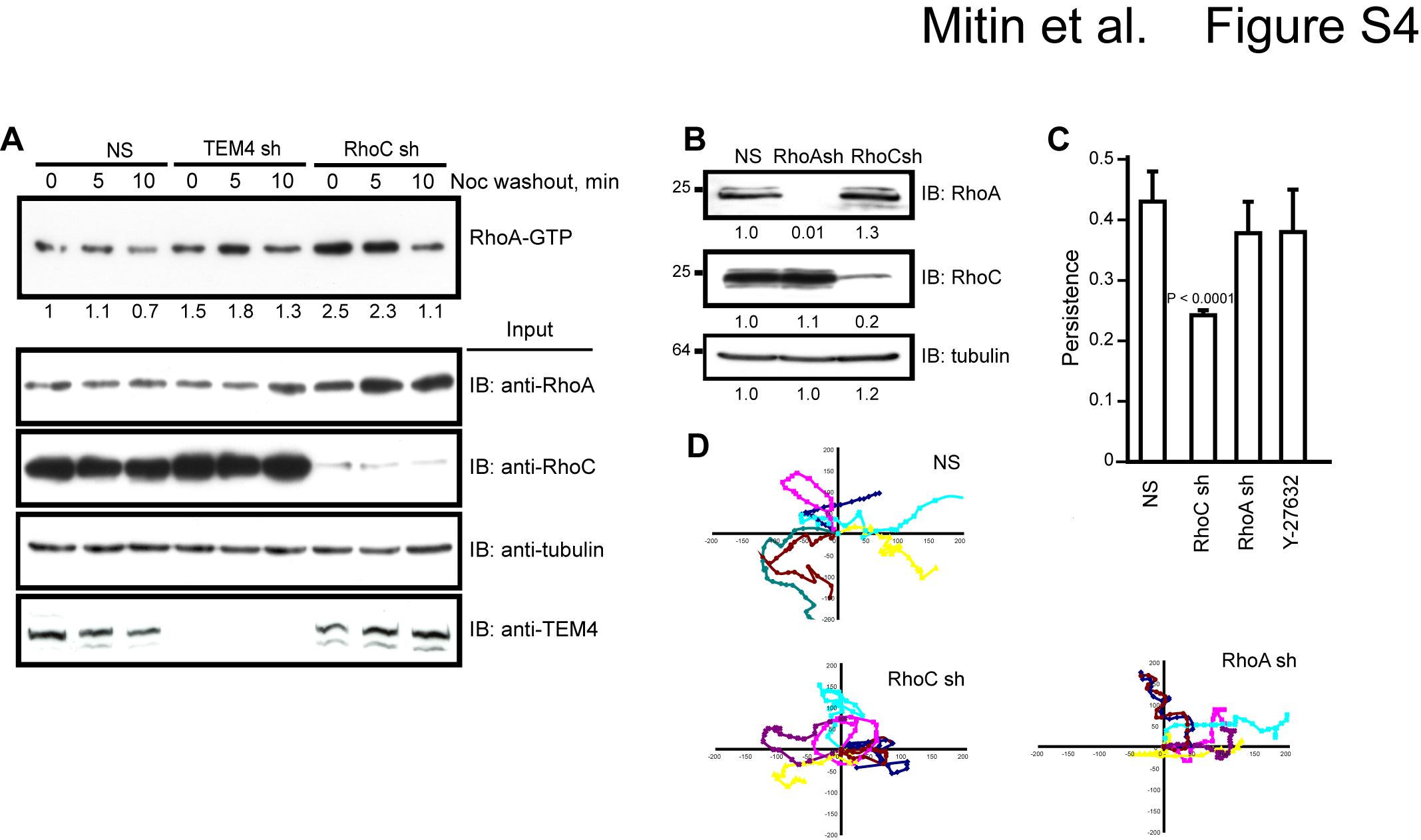

Supplement: Figure S4 — TEM4 and RhoC antagonize activation of RhoA. (A) Knockdown of TEM4 or RhoC promotes activation of RhoA. Cells depleted of TEM4 or RhoC or NS control were left untreated (GM), treated with nocodazole (Noc) or treated with nocodazole with subsequent nocodazole washout. Active RhoA was pulled down in GTPase pull-down assay and levels of active and total RhoA were determined by western blot analysis. (B) Western blot confirming knockdown of RhoA and RhoC expression levels by lentivirus-based RNAi constructs in cells used for single cell tracking. NS; non-specific shRNA. (C) Persistence of two-dimensional cellular migration of HUVECs expressing NS, RhoC or RhoA shRNAs or treated with Y-27632. (D) Wind-Rose plots depicting migratory tracks of six individual migrating cells in each experimental group. Values on x and y scales are arbitrary. (TIF) [file pone.0066260.s004.tif]

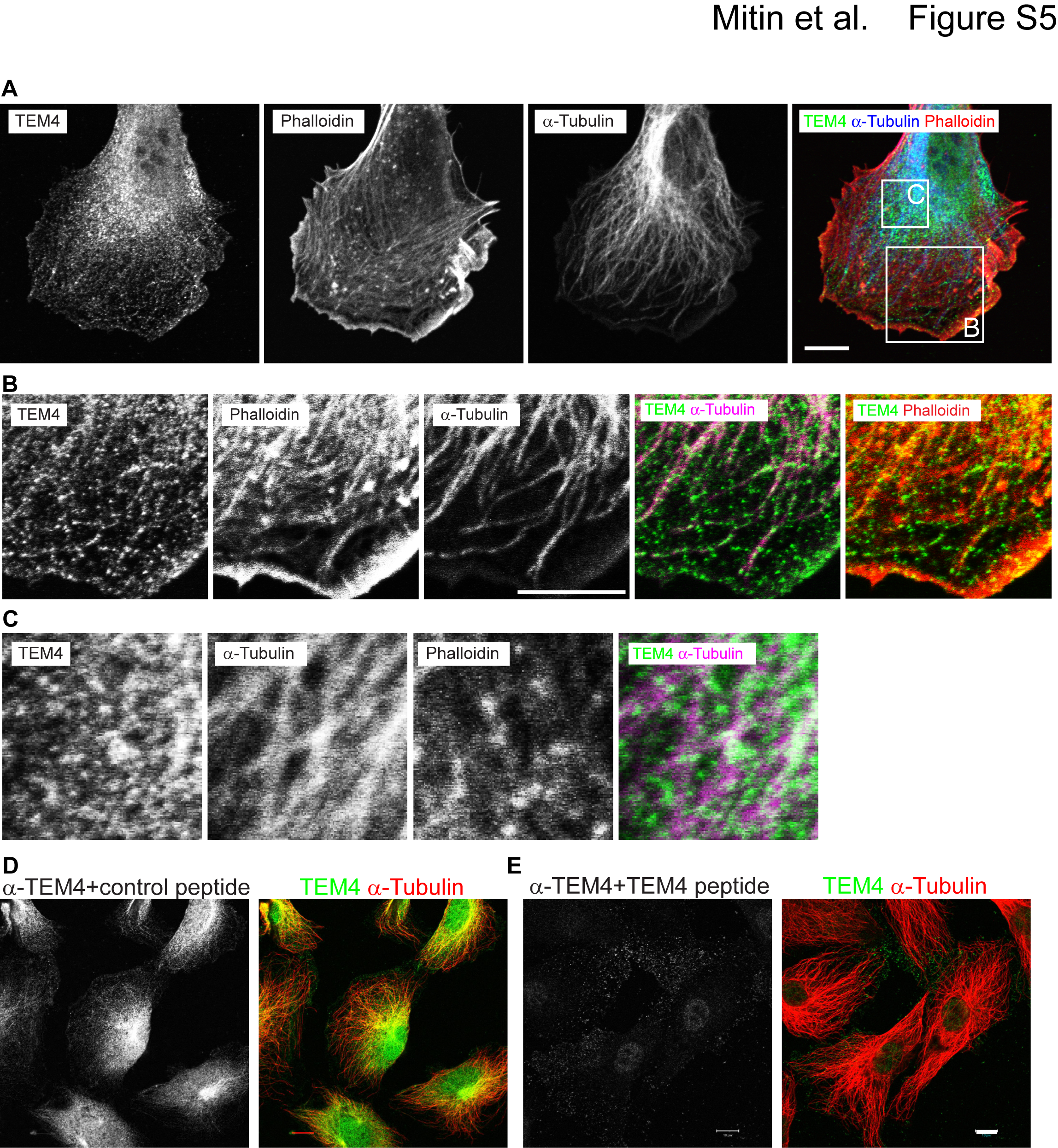

Supplement: Figure S5 — Localization of endogenous TEM4 to actin filaments and microtubules in protrusive areas of the cell. (A) HUVECs were stained with antibodies against TEM4 and α-tubulin and Alexa-594 phalloidin. The close-up of cell periphery (B) or cell body (C) is shown. Specificity of TEM4 staining was confirmed by preincubating the TEM4 antibody with TEM4 immunizing peptide (E) or control peptide (D) of similar length. Scale bar 10 µm. (TIF) [file pone.0066260.s005.tif]
