# Supplementary material for: The RhoGEF TEM4 Regulates Endothelial Cell Migration by Suppressing Actomyosin Contractility
Source: PLoS One. 2013 Jun 18;8(6):e66260. doi: 10.1371/journal.pone.0066260 (PMC3688894; doi:10.1371/journal.pone.0066260)
Supplement: Results S1 — (DOCX) [file pone.0066260.s015.docx]

**Supplemental Results**

**Specificity of BiFC biosensors**

To validate the specificity of the RhoC-BiFC probe, we compared BiFC of wild type RhoC constructs to point mutants that impair the RhoC-ROCK interaction (RhoC 68A or ROCK RBD N1004A) [31,78]. 293T cells were cotransfected with plasmids encoding each fusion protein pair (Fig. S2 A, B) in combination with a plasmid encoding red fluorescent protein tRFP, and the Venus/tRFP emission ratio was calcu­lated. Coexpression of the wild-type fusion proteins in cells resulted in fluores­cence comple­mentation (Fig. S2 C, D). In contrast, expression of mutants of the ROCK RBD or RhoC significantly decreased complementation suggesting that BiFC signal is specific for active RhoC.

To further validate RhoC-BiFC sensor, we expressed BiFC sensor at a level comparable to endogenous RhoC in HUVECs (Fig. S3 B) and visualized the BiFC-derived fluorescent signal. As shown in Fig. S3A, activation of RhoC was readily detected in protrusive areas of cells. While low levels of GFP-RhoC fusion protein or GFP alone were detected in cellular protrusions, no accumulation of fluorescence at the leading edge was detected (Fig. S3 A, arrows), suggesting that the observed changes in RhoC-BiFC signal reflect activation of RhoC in membrane protrusions, which is consistent with the RhoC activation profile observed using a FRET-based RhoC biosensor [27]. More importantly, knockdown of TEM4 severely impaired activation of RhoC in membrane protrusions (Fig. S3D). Therefore, we concluded that the BiFC-based sensor can be used to visualize activation of RhoC in cells and test the *in vivo* substrate specificity of RhoGEFs.
